# Effects of dietary inorganic and organic iron sources on maternal and piglet iron status across one complete reproductive cycle in highly prolific sows

**DOI:** 10.1093/tas/txaf080

**Published:** 2025-06-14

**Authors:** Katlyn A McClellan, Abigail L Morse, Yanbin Shen, Joe Crenshaw, Joshua Jendza, Eric M Weaver

**Affiliations:** Department of Animal Science, South Dakota State University, Brookings, SD 57007, USA; Department of Animal Science, South Dakota State University, Brookings, SD 57007, USA; APC LLC, Ankeny, IA 50021, USA; APC LLC, Ankeny, IA 50021, USA; QualiTech, LLC, Chaska, MN 55318, USA; Department of Animal Science, South Dakota State University, Brookings, SD 57007, USA

**Keywords:** anemia, polysaccharide iron complexes, ferrous sulfate, heme iron, sows, suckling pigs

## Abstract

Ferrous sulfate (FeSO_4_) is commonly used in swine diets, yet sow anemia remains prevalent, prompting the need to evaluate alternative iron sources. This study evaluated polysaccharide complexed iron (PC-Fe; non-heme organic iron source) and spray-dried bovine blood cells (HEME-Fe; heme-organic iron source) as alternatives to FeSO_4_ (CON-Fe; inorganic iron source) on sow and offspring iron status. At 2 ± 1 d of gestation, 44 female pigs were allocated by parity (0 to 4), body weight, and initial blood hemoglobin (Hb) to one of three dietary iron treatments: a control group (CON-Fe) receiving 125 mg/kg of iron from FeSO_4_ monohydrate (n = 15), a non-heme organic iron group (PC-Fe) receiving 125 mg/kg iron from a polysaccharide-complexed iron source (n = 15), and a heme iron group (HEME-Fe) receiving equal amounts of Fe from FeSO_4_ and organic heme iron from spray-dried red blood cells (RBCs) (n = 14). Sows were fed their assigned diets until weaning, meeting or exceeding the estimated nutrient requirements for gilts during both gestation and lactation. Sow blood was collected on days 0, 30 ± 2, 60 ± 2, 90 ± 2, and 110 ± 2 of gestation, and at weaning (day 18 ± 3 of lactation). In piglets, Hb was measured within 12 hours after birth and at weaning (day 18 ± 3) using a point-of-care analyzer (HemoCue 201+). Additionally, four piglets per litter were sampled at birth and two at weaning for serum analyses via jugular puncture. Sow and piglet serum samples were analyzed for serum iron (SI), ferritin (SF), and total iron-binding capacity (TIBC). Sow whole blood was also analyzed for hematological parameters including white blood cells, RBC, Hb, packed cell volume, and platelet counts. On day 90 of gestation, sows supplemented with HEME-Fe had higher Hb concentrations (12.1 g/dL) compared to those receiving CON-Fe (10.9 g/dL) and PC-Fe (10.8 g/dL) (*P* = 0.001). On day 30, PC-Fe sows had higher SF concentrations (21.4 ng/mL) than CON-Fe sows (10.4 ng/mL; *P* = 0.027), with HEME-Fe sows showing intermediate values (15.4 ng/mL). The PC-Fe sows also tended to have lower TIBC (576.7 µg/dL) than CON-Fe sows (613.1 µg/dL; *P* = 0.059), with HEME-Fe again being intermediate (600.4 µg/dL). Piglet anemia at birth (Hb < 10 g/dL) was lowest in PC-Fe (29%), followed by HEME-Fe (42%), both lower than CON-Fe (75%) (*P* < 0.001). PC-Fe improved early maternal iron storage, while HEME-Fe supported late gestational Hb, both enhancing neonatal iron status compared to FeSO_4_.

## Teaser

Supplementing sows with organic iron improved maternal iron status during gestation and reduced anemia prevalence in newborn piglets. Enhancing maternal iron transfer through organic dietary iron sources may help optimize neonatal piglet iron status.

## INTRODUCTION

Iron is essential for numerous physiological functions, including growth, reproduction, and immune competence. Hemoglobin (Hb), the primary oxygen transport protein in red blood cells (RBCs), requires iron for its synthesis (Farid et al., 2023). In sows, iron also plays a vital role in supporting embryonic and fetal development. As gestation advances—particularly during late pregnancy—and throughout lactation, maternal iron requirements increase due to accelerated fetal iron accretion and the demands associated with neonatal growth (Mahan et al., 2009). Although higher reproductive performance is linked to increased mineral losses (Mahan and Newton, 1995), the extent and efficiency of iron redistribution and fetal transfer in modern highly prolific sows remain poorly defined.

Iron deficiency can result in anemia (Clark, 2008), which is a growing concern in pregnant sows. Anemia, commonly defined as Hb < 10 g/dL (Castevens et al., 2020; Noblett et al., 2021), is frequently observed in late gestation and lactation, with risk increasing as parity advances (McClellan et al., 2024a). Anemic sows are at greater risk for farrowing complications, prolonged parturition, and stillbirths (Bhattarai et al., 2018; McClellan et al., 2024b). However, the extent to which anemia reflects true iron deficiency remains unclear, due in part to limited data on maternal iron biomarkers and the lack of established reference ranges for key indicators of iron metabolism.

Ferrous sulfate has traditionally served as the primary oral iron supplement in swine diets (Kegley et al., 2002), though its bioavailability may be reduced under conditions that hinder mineral absorption (Henry and Miller, 1995). The absorption and activity of inorganic iron are impaired by the antagonism among trace elements and macro-elements (Ulbreit, 2005). In contrast, organic iron sources may enhance absorption by reducing antagonistic interactions within the gastrointestinal tract (Liang et al., 2024). In pregnant sows, supplementation with organic trace minerals, including iron, has been associated with improved piglet outcomes, including increased birth and weaning weights, reduced stillbirth and pre-weaning mortality rates, and enhanced neonatal iron status (Close, 1998, 1999).

Although organic trace minerals are not a new concept, most prior research on organic iron supplementation in sows has focused on reproductive outcomes and piglet iron status (Close, 1998, 1999; Wang et al., 2014). Fewer studies have evaluated the effects of organic iron on maternal iron status specifically. The present study aimed to compare the effects of three dietary iron sources—ferrous sulfate, polysaccharide-complexed iron, and heme iron on reproductive performance and iron status in sows and their offspring across a single reproductive cycle.

## MATERIALS AND METHODS

### Animal Care and Use

All procedures used in this study were approved by the South Dakota State University (SDSU) Institutional Animal Care and Use Committee and adhered to the Guide for the Care and Use of Agricultural Animals in Research and Teaching (American Dairy Science Association et al., 2020). The animals used in this experiment were raised and managed at the SDSU Swine Education and Research Facility, located in Brookings, South Dakota.

### Animals Used and Housing

A total of 44 female pigs (PIC Camborough 42) of parities 0 to 4 (average parity: 1.5) and their offspring (n = 639) were used in this experiment from breeding (day 0 of gestation) to weaning (18 ± 3 of lactation). For this study, parity 0 was defined as females in their first gestation that had not yet farrowed. All females were housed in gestation stalls (0.61 m × 1.98 m) from breeding until around day 110 of gestation, where they were relocated to farrowing crates (1.83 m × 2.43 m) until weaning.

### Dietary Treatments and Feeding

The females were assigned to one of three dietary iron treatments based on parity, body weight (BW), and Hb 2 ± 1 d after breeding, with each treatment group consisting of 14 to 15 females. The control group (CON-Fe) received 125 mg/kg of iron from ferrous sulfate monohydrate (FeSO_4_; 30% iron) (n = 15). The non-heme organic iron group (PC-Fe) received 125 mg/kg of iron from a polysaccharide-complexed iron source (14.6% iron) (n = 15). The heme iron group (HEME-Fe) receiving equal amounts of iron from FeSO_4_ and organic heme iron from spray dried beef RBCs (0.27% iron), with each source contributing 62.5 mg/kg of iron (n = 14). All three experimental diets were formulated to have a similar nutrient composition (Table 1) and to meet or exceed the nutrient requirements for gilts expected to produce litters of 14 piglets (NRC, 2012).

**Table 1.** Composition of gestation and lactation diets (as-fed basis)

Item	Gestation			Lactation		
Ingredient, %	CON-Fe	PC-Fe	HEME-Fe	CON-Fe	PC-Fe	HEME-Fe
Ground Corn	82.38	82.38	84.93	59.45	59.45	62.32
Soybean meal	7.73	7.73	2.73	30.15	30.15	25.29
Soybean hulls	6.18	6.18	6.18	5.00	5.00	5.00
Monocalcium phosphate 21%	1.29	1.29	1.41	0.59	0.59	0.69
Limestone	1.21	1.21	1.30	1.46	1.46	1.45
Salt	0.50	0.50	0.50	0.50	0.50	0.50
Trace mineral premix^1^	0.25	0.25	0.25	0.25	0.25	0.25
L-Lysine, HCL 99%	0.10	0.10	0.02	0.06	0.06	0.00
Vitamin Premix^2^	0.05	0.05	0.05	0.05	0.05	0.05
Rabon 7.76^3^	0.13	0.13	0.13	0.13	0.13	0.13
Swine Toxin Binder^4^	0.10	0.10	0.10	0.10	0.10	0.10
Phytase	0.08	0.08	0.08	0.08	0.08	0.08
L-Valine	0.00	0.00	0.00	0.03	0.03	0.00
DL- Methionine—99%	0.00	0.00	0.00	0.01	0.01	0.03
Spray dried bovine red blood cells^5^	0.00	0.00	2.31	0.00	0.00	2.31
Calculated composition						
Dry matter, %	89.5	89.5	89.5	89.50	89.50	89.50
Metabolizable energy, kcal/kg	3,179.0	3,179.0	3,179.0	3,300.0	3,300.0	3,300.0
Crude protein, %	11.00	11.00	11.00	19.80	19.80	19.80
Calcium, %	0.83	0.83	0.83	0.89	0.89	0.89
Phosphorus-total, %	0.55	0.55	0.55	0.50	0.50	0.50
Phosphorus -dig, %	0.38	0.38	0.38	0.37	0.37	0.37
SID amino acids, %						
Lysine	0.55	0.55	0.55	0.97	0.97	0.97
Threonine	0.42	0.42	0.43	0.29	0.29	0.30
Methionine	0.21	0.21	0.21	0.63	0.63	0.63
Tryptophan	0.09	0.09	0.09	0.22	0.22	0.22
Isoleucine	0.36	0.36	0.28	0.73	0.73	0.66
Valine	0.50	0.50	0.54	0.83	0.83	0.89
Arginine	0.70	0.70	0.70	1.17	1.17	1.17
Histidine	0.33	0.33	0.33	0.47	0.47	0.47
Leucine	1.20	1.20	1.20	1.55	1.55	1.55
Phenylalanine	0.59	0.59	0.59	0.85	0.85	0.85
Analyzed composition						
Crude protein, %	10.4	10.1	10.0	18.1	18.1	18.2
Crude fat, %	2.4	2.3	2.6	4.0	4.0	4.1
Crude fiber, %	3.4	3.3	3.2	3.0	3.0	2.9
Ash, %	4.3	4.3	4.5	4.6	4.8	4.5
Iron, mean (SD) mg/kg^6^	246 (36.2)	244 (27.8)	241 (31.8)	267 (55.3)	259 (47.8)	260 (68.0)

^¹^Minimum levels provided per kg of diet: Copper (15 mg, J & R Distributing Inc); Iodine (0.36 mg, J & R Distributing Inc); Iron—CON-Fe (125 mg ferrous sulfate, QualiTech LLC); PC-Fe (125 mg SQM iron, QualiTech LLC); HEME-Fe (62.5 mg ferrous sulfate, QualiTech LLC); Manganese (40 mg, J & R Distributing Inc); Selenium (0.3 mg, J & R Distributing Inc); Zinc (156 mg, J & R Distributing Inc).

^2^Minimum supplied the following per kilogram of diets: Vitamin A 11,000 IU, Vitamin D3 1,650 IU, Vitamin E 55 IU; Vitamin B12 0.044 mg, Menadione 4.4 mg, Biotin 0.165 mg, Folic Acid 1.1 mg, Niacin 55 mg, d-Pantothenic Acid 60.5 mg, Vitamin B6 6.3 mg, Riboflavin 9.9 mg, Thiamin 3.3 mg (J & R Distributing Inc).

^3^Minimum supplied the following per kilogram of diets: Active Ingredient: Tetrachlorovinphos 75.9 mg (Elanco US Inc.).

^4^Algonite; blend of dried yeast cells, diatomaceous earth, and algae (Olmix NA Inc.).

^5^AP301 (APC LLC).

^6^Analyzed iron represents the mean of three subsamples within a given diet.

In gestation, females were fed once per day approximately 2.0 to 2.6 kg of feed. Feed allowances during gestation were adjusted based on parity to ensure nutrient needs were met, with revisions every 30 d according to body condition scoring and visual assessments (e.g., thin, ideal condition, or over-conditioned). Once the females were moved to farrowing crates at day 110 of gestation, they were provided 2.7 kg/d of lactation feed distributed in six meals from 5 a.m. to 8 p.m. using an electronic feeding system (Gestal Solo, Jyga Technologies Inc.). After farrowing, sows were given ad libitum access to lactation feed until weaning.

Daily iron intake was calculated based on daily feed intake and analyzed dietary iron content. Dietary iron content was determined from the mean (SD) of three analyzed subsamples per treatment collected across the gestation period (246 ± 36.2, 244 ± 27.8, and 241 ± 31.8 mg/kg of iron for CON-Fe, PC-Fe, and HEME-Fe, respectively) and three analyzed subsamples per treatment collected across the lactation period (267 ± 55.3, 259 ± 47.8, and 260 ± 68.0 mg/kg of iron for CON-Fe, PC-Fe, and HEME-Fe, respectively). The total heme iron content of the heme source used (spray dried bovine RBCs) was analyzed from three subsamples (mean of 2,186 ± 50.1 mg/kg of iron). The determination of heme iron content was based on the difference spectra of pyridine hemochromogen (Sinclair et al., 1999).

### Sow Sampling and Processing

Throughout the experiment, females were weighed and had blood samples collected on days 0 (breeding day), 30 (± 2), 60 (± 2), 90 (± 2), and 110 (± 2) days after breeding, as well as on the day of weaning, which occurred at 18 (± 3) days of lactation. Additionally, females were weighed on day 2 of lactation. For blood sampling, the females were restrained using a snare, and blood was withdrawn from the jugular vein using 18 gauge × 1½ in. needles (aluminum hub needle; Animal Health International, Loveland, CO). Blood samples were collected into two types of tubes: 6 mL EDTA tubes (Greiner Bio-One VACUETTE™ K3EDTA Blood Collection Tubes; Fisher Scientific, Waltham, Massachusetts) for whole blood, and 6 mL plain tubes (BD Vacutainer™ Venous Blood Collection Tubes: Vacutainer Plus™ Plastic Serum Tubes, Silicone-Coated, with Hemogard™ Closure; Fisher Scientific, Waltham, Massachusetts) for serum. All blood samples were collected in the morning after feeding to minimize the effects of diurnal variation. Sampling times were standardized across days to reduce variability related to feeding status.

Colostrum (50 mL per sow) was collected from all teats after the birth of the first piglet (on day 0 of lactation), before the first piglet suckled. The pooled colostrum sample was immediately combined with 4 microliters of a milk stabilizer (Bronolab Liquid; Advanced Instruments LLC., Norwood, MA) and stored at − 20°C until further analysis. Placental tissue samples were collected for each sow, extracting a sample from the same location on each placental mass, and pooling each sample together. Placental tissue samples were immediately snap frozen using liquid nitrogen, and then stored at − 20°C until further analysis for trace mineral concentrations.

### Reproductive Performance Data

To monitor the farrowing process, surveillance cameras (Reolink, New Castle, Delaware) were employed, complemented by human observation (checked hourly by trained research technicians and barn staff). Farrowing assistance by sleeving, followed barn protocol. Sleeving was employed when 60 min had passed since the birth of the previous piglet. When sleeved by a research technician, if a piglet was felt in the birth canal, it was pulled. One piglet was attempted to be retrieved during each sleeve, unless more than one piglet was present in the birth canal, in which case both were pulled. The research technician did not sleeve again until 60 min elapsed again. Farrowing duration was defined as the interval of time elapsed between the birth of the first and last piglet of the litter. Identification of stillborn piglets was done using a lung flotation technique (Bhattarai et al., 2018).

### Piglet Sampling and Processing

All piglets were weighed within 12 hours after birth and again at weaning (18 ± 3 d of age). Blood samples were collected from four piglets per litter—one small, two average-sized, and one large piglet (n = 176)—within 12 hours of birth and prior to iron dextran administration. Immediately following blood collection, all piglets received a 200 mg intramuscular injection of iron dextran on day 1 of age. Standard processing procedures, including tail docking and castration of male piglets, were performed within 3 d after birth. At weaning, blood samples were collected from the two average-sized piglets per litter (n = 88). Blood was drawn via jugular vein puncture using 21 gauge × ½ in. aluminum hub needles (Animal Health International, Loveland, CO) into 6 mL serum tubes (BD Vacutainer™ Venous Blood Collection Tubes: Vacutainer Plus™ Plastic Serum Tubes, silicone-coated, with Hemogard™ closure; Fisher Scientific, Waltham, MA). Hemoglobin concentrations were measured at both timepoints (within 12 hours after birth and at weaning) for each piglet using a point-of-care testing method. A 20 gauge × 1 in. needle was used to prick the ear vein, and a droplet of blood was loaded into disposable microcuvettes via capillary action. Microcuvettes were analyzed using the HemoCue Hb 201 + device (HemoCue America, Brea, CA), which provided Hb readings within 60 s. The HemoCue system has been validated for use in piglets, with Hb values shown to be within 1% of laboratory-based analyses for both ear and jugular vein samples (McClellan et al., 2025).

### Analytic Methods

The EDTA whole blood samples from the females were kept at room temperature and placed immediately on a blood rotator to ensure continuous, gentle mixing until analysis. Samples were analyzed within 3 ± 2 hours of collection to assess hematological parameters, including Hb concentration, RBCs, white blood cell count (WBC), packed cell volume (PCV), and platelet (PLT) count. All hematological analyses were conducted using the Siemens Advia 2120/2120i Hematology System (Siemens Healthcare Diagnostics, Eschborn, Germany), which employs the standard hemiglobincyanide (HiCN) method for Hb quantification.

Blood samples from the females and piglets were allowed to clot at room temperature for 1 hour after collection, then centrifuged at 2,000 × g for 20 min. Serum was separated and stored at − 20°C until analysis. Serum iron (SI), serum ferritin (SF), and total iron-binding capacity (TIBC) were quantified using the Vet AXCEL Clinical Chemistry System.

Colostrum and placental tissue samples were analyzed for iron, copper, and zinc concentrations. Colostrum samples were weighed and digested overnight in a 95°C oven using approximately twice the sample mass of nitric acid. Following digestion, samples were diluted with deionized water to 25 times the original sample mass. Placental tissue samples were sectioned and digested overnight at 95°C using approximately 10 times the dry tissue mass of nitric acid. When sufficient tissue was available, a separate section was dried in a 75°C oven overnight to determine the dry matter fraction for calculating dried tissue mass. If the sample was too small for a separate drying step, the same tissue section was dried prior to digestion. Digested placental samples were then diluted with deionized water to 100 times the dried tissue mass.

Elemental analysis of colostrum and placental tissue followed the method described by Wahlen et al. (2005) using an Agilent 7900 Inductively Coupled Plasma Mass Spectrometer (ICP-MS). An aliquot of each diluted tissue digest and calibration standard was further diluted 25-fold with a solution containing 0.5% EDTA, Triton X-100, 1% ammonium hydroxide, 2% 1-butanol, and internal standards including 5 ppb scandium, and 7.5 ppb germanium, rhodium, indium, and bismuth. The ICP-MS was tuned to achieve a minimum sensitivity of 7500 cps for 1 ppb yttrium (mass 89), a maximum oxide level of 1.0% (determined by the 156/140 mass ratio), and less than 2.0% double-charged ions (determined by the 70/140 mass ratio).

### Statistical Analysis

Data were assessed for homogeneity of variances and normality of distribution. The PROC MIXED procedure in SAS 9.4 (SAS Institute Inc., Cary, NC) was used to analyze blood parameters of females and piglets across treatments at each time point, as well as sow BW and reproductive performance. Weaning age was included as a covariate in the analysis of individual piglet BW and litter weight at weaning to account for variability in weaning age. Treatment differences were evaluated using Tukey’s honest significant difference test. The percentages of stillborn and mummified piglets were calculated by dividing the number of stillborn or mummified piglets by the total number of piglets born. Females and piglets with Hb < 10 g/dL were categorized as anemic (Castevens et al., 2020; Noblett et al., 2021; McClellan et al., 2024a, b). The proportions of anemic and non-anemic animals within each treatment group were compared using chi-square tests. The alpha of ≤ 0.05 determined significance and an alpha of >0.05 ≤0.10 was considered a tendency.

## RESULTS

### Sow Reproductive Performance and Feed Intake

There were no treatment differences in sow BW or average daily feed intake during gestation or lactation. Additionally, no differences in estimated daily iron intake were observed between treatments during gestation and lactation (Table 2).

**Table 2.** Sow measurements by iron source treatment

Item	CON-Fe	PC-Fe	HEME-Fe	SEM	*P*-value
No. sows, n	15	15	14	-	-
Avg. sow parity, n	1.5	1.5	1.5	-	-
Sow body weight, kg					
Gestation					
d0	199.6	200.2	199.3	10.9	0.963
d30	205.1	203.9	204.4	7.1	0.988
d60	219.1	220.3	217.7	6.7	0.955
d90	240.1	240.6	238.4	6.4	0.967
d110	262.9	263.0	258.9	5.6	0.833
Lactation					
d2	243.4	242.3	243.4	7.7	0.542
d18	244.9	248.9	242.2	8.1	0.823
Gestation feed intake, kg/d	2.4	2.4	2.3	0.1	0.335
Gestation iron intake mg/d^1^	585.1	572.9	573.1	20.6	0.874
Lactation feed intake kg/d	6.6	7.0	6.4	0.32	0.256
Lactation iron intake mg/d^1^	1815.4	1855.8	1731.3	86.5	0.488

^1^Caclulated as individual feed intake × analyzed diet iron content. Analyzed iron represents the mean of three subsamples within a given phase.

Farrowing duration was similar across all treatments with no differences observed in the total number of pigs born or live born, nor in the rates of stillborn or mummified piglets (Table 3). Piglet BW at birth tended to be greater in the PC-Fe group than in the CON-Fe group, while HEME-Fe piglets showed intermediate birth weights (*P* = 0.096). No differences were found between treatments in total litter weight at birth, nor in weaned piglet BW or total weaned litter weight. Sows in the PC-Fe group tended to wean more piglets than the HEME-Fe group, with the CON-Fe group being intermediate (*P* = 0.102). However, the proportion of pigs weaned was similar between the PC-Fe and HEME-Fe groups, with PC-Fe sows weaning a greater proportion weaned than CON-Fe sows (*P* = 0.024). Age at weaning tended to be greater in the CON-Fe group than in the PC-Fe group, with the HEME-Fe group being intermediate (*P *= 0.099).

**Table 3.** Reproductive performance by iron source treatment

Item	CON-Fe	PC-Fe	HEME-Fe	SEM	*P*-value^1^
No. sows, n	15	15	14	-	-
Farrowing duration^2^, min	319.50	313.07	343.50	53.37	0.898
Total born, n	17.93	17.71	17.33	0.84	0.854
Live born, n	15.93	16.29	15.20	0.57	0.354
Stillborn^3^, %	7.50	5.53	5.35	2.22	0.653
Mummy^4^, %	1.50	1.05	2.34	1.01	0.627
Pig weight, kg					
d0	1.32^x^	1.50^y^	1.44^xy^	0.07	0.096
d18^5^	5.75	5.66	5.28	0.18	0.142
Litter weight, kg					
d0	19.47	21.10	18.81	1.27	0.153
d18^5^	76.20	77.11	74.02	4.96	0.863
Pigs weaned, n	13.80^xy^	14.79^x^	13.50^y^	0.49	0.102
Pigs weaned, %	89.88^b^	95.08^a^	90.57^ab^	2.55	0.024
Wean age, d	19.00^x^	17.87^y^	18.14^xy^	0.4	0.099

^1^Means within the same column that do not share the same lower-case superscript are different. ^a,b^ Represents comparisons made at *P* < 0.05 and ^x,y^ represents comparisons made at *P *< 0.10 across treatment within day.

^2^Farrowing duration adjusted for total born as a covariate; one sow in the CON-Fe group was excluded from this analysis due to an outlier value (>3-fold the group standard deviation).

^3^The stillborn rate is expressed as a percentage of stillborn piglets relative to the total number of piglets born.

^4^The mummy rate is expressed as a percentage of mummified piglets relative to the total number of piglets born.

^5^Wean body weights and litter weights adjusted for wean age as a covariate.

### Sow Hematological Parameters and Iron Indicators

Sow Hb did not differ between treatments on days 0, 30, or 60 of gestation (Table 4). However, on day 90 of gestation, HEME-Fe sows had higher Hb than both CON-Fe and PC-Fe sows (*P* = 0.001). No differences in sow Hb were observed between treatments on day 110 of gestation or day 18 of lactation. There were no differences in WBC, RBC, PCV, or PLT count between treatments at any time point.

**Table 4.** Hematological parameters in sows by iron source treatment and day^1^

Experimental Groups^2^	Gestational Days					Lactation Day
	Day 0	Day 30	Day 60	Day 90	Day 110	Day 18
	**Hemoglobin concentration, g/dL**					
CON-Fe	10.3	11.8	12.0	10.9^b^	10.3	9.4
PC-Fe	10.4	11.5	12.2	10.8^b^	10.4	10.2
HEME-Fe	10.4	11.6	12.8	12.1^a^	10.4	9.9
**SEM**	0.4	0.4	0.3	0.3	0.4	0.3
***P*-value**	0.959	0.851	0.157	0.001	0.910	0.133
	**Red blood cell count, m/uL**					
CON-Fe	5.8	6.6	6.6	6.0	6.1	5.2
PC-Fe	5.8	6.3	6.3	6.0	6.0	5.3
HEME-Fe	5.7	6.4	6.5	6.2	5.7	5.3
**SEM**	0.3	0.2	0.2	0.2	0.3	0.2
***P*-value**	0.918	0.694	0.315	0.613	0.508	0.892
	**White blood cell count, k/uL**					
CON-Fe	15.1	13.5	12.0	12.5	11.7	14.2
PC-Fe	13.7	13.3	12.1	11.1	12.3	13.9
HEME-Fe	13.9	13.1	12.0	12.0	10.8	14.2
**SEM**	1.0	0.8	0.7	0.8	0.8	1.1
***P*-value**	0.562	0.934	0.964	0.351	0.375	0.961
	**Packed cell volume, %**					
CON-Fe	36.0	39.8	40.0	38.0	37.3	34.4
PC-Fe	35.4	37.5	38.4	36.5	37.6	35.7
HEME-Fe	35.5	38.6	39.3	37.3	37.1	34.0
**SEM**	1.1	1.5	0.8	0.7	0.9	0.8
***P*-value**	0.952	0.398	0.205	0.152	0.998	0.118
	**Platelets, k/uL**					
CON-Fe	285.5	241.2	196.5	228.9	170.3	288.8
PC-Fe	279.9	195.8	202.6	216.9	189.1	262.1
HEME-Fe	291.3	221.2	199.6	234.9	195.8	269.2
**SEM**	23.2	24.2	13.4	22.5	16.6	20.5
***P*-value**	0.976	0.203	0.927	0.596	0.734	0.327

^1^Means within the same column that do not share the same lower-case superscript are different. ^a,b^ Represents comparisons made at *P* < 0.05 across treatment within day.

^2^Number of observations per treatment group: CON-Fe = 15, PC-Fe = 15, HEME-Fe = 14.

No differences in total SI were observed between treatments at any time point (Table 5). On day 30 of gestation, PC-Fe sows had higher SF levels than CON-Fe sows, with HEME-Fe sows showing intermediate values (*P* = 0.027). Serum ferritin levels did not differ by treatment at any other time point. For TIBC, differences were only observed on day 30 of gestation, where PC-Fe sows tended to have lower TIBC levels than CON-Fe sows, with HEME-Fe sows showing intermediate values (*P* = 0.059). No differences in TIBC were observed at any other timepoint between the treatments.

**Table 5.** Serum iron status in sows by iron source treatment and day^1^

Experimental Groups^2^	Gestational Days					Lactation Day
	Day 0	Day 30	Day 60	Day 90	Day 110	Day 18
	**Serum iron, ug/dL**					
CON-Fe	96.3	111.6	130.5	106.6	59.6	114.6
PC-Fe	101.8	121.9	119.2	102.2	63.3	106.8
HEME-Fe	85.1	120.0	127.4	101.0	65.7	110.0
**SEM**	8.5	6.8	8.0	6.9	10.2	12.0
***P*-value**	0.352	0.334	0.564	0.824	0.854	0.890
	**Serum ferritin, ng/mL**					
CON-Fe	13.3	10.4^b^	19.2^y^	20.2	18.9	8.0
PC-Fe	11.8	21.4 ^a^	31.2^x^	27.4	20.6	10.3
HEME-Fe	11.0	15.4 ^ab^	27.8 ^xy^	28.3	20.8	18.7
**SEM**	2.4	3.9	4.8	4.5	2.6	8.8
***P*-value**	0.707	0.027	0.144	0.338	0.838	0.389
	**Serum total iron binding capacity, µg/dL**					
CON-Fe	542.2	613.1^y^	630.0	586.5	495.5	566.2
PC-Fe	556.2	576.7^x^	620.6	522.5	494.0	550.1
HEME-Fe	514.5	600.4^xy^	641.3	554.8	480.7	563.3
**SEM**	18.6	11.3	18.4	17.7	50.5	28.7
***P*-value**	0.265	0.059	0.716	0.213	0.879	0.908

^1^Means within the same column that do not share the same lower-case superscript are different. ^a,b^ Represents comparisons made at *P* < 0.05 and ^x,y^ represents comparisons made at *P *< 0.10 across treatment within day.

^2^Number of observations per treatment group: CON-Fe = 15, PC-Fe = 15, HEME-Fe = 14.

### Sow Anemia Prevalence

**Figure 1.**
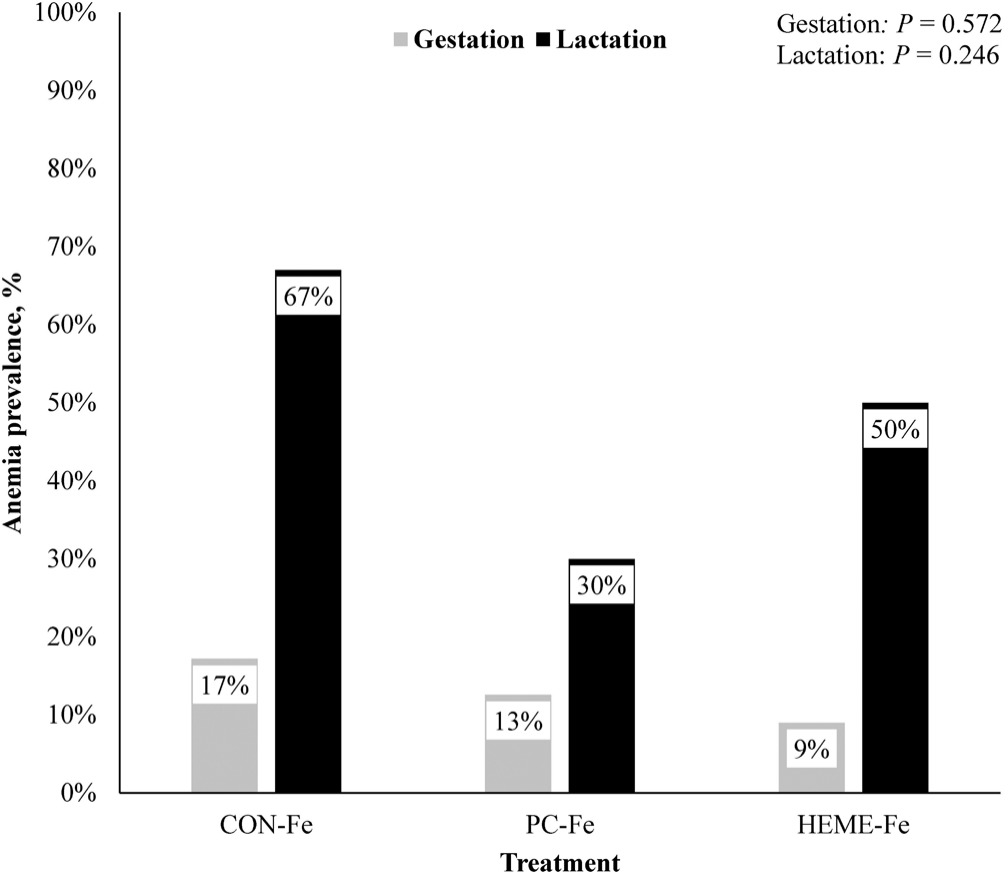
Sow anemia prevalence across gestation and at weaning by iron source treatment Anemia is defined as hemoglobin < 10 g/dL. Prevalence across gestation was assessed at four timepoints: days 30, 60, 90, and 110. Anemia prevalence during lactation was evaluated at weaning which occurred on day 18 of lactation.

When assessing the prevalence of anemia (defined as Hb < 10 g/dL) across gestational timepoints (days 30, 60, 90, and 110) by treatment, the prevalence was 17% in the CON-Fe sows, 13% in the PC-Fe sows, and 9% in the HEME-Fe sows (Figure 1). However, no difference in prevalence was observed between treatments (*P* = 0.572). At weaning, the prevalence of anemia was 67% in the CON-Fe sows, 30% in the PC-Fe sows, and 50% in the HEME-Fe sows. Additionally, no difference in prevalence was observed between treatments at weaning (*P* = 0.246).

### Piglet Hb and Iron Indicators by Treatment and by Day

At birth and weaning, no differences were observed in piglet Hb, SI, SF, or TIBC between treatments at either time point (Table 6).

**Table 6.** Piglet hemoglobin concentration and iron indicators by iron source treatment and day

Experimental Groups^1^	Days of age	
	Day 1	Day 18
	**Hemoglobin concentration, g/dL**	
CON-Fe	10.8	10.8
PC-Fe	11.0	10.9
HEME-Fe	10.3	10.8
**SEM**	0.3	0.1
***P*-value**	0.196	0.310
	**Serum iron, µg/dL**	
CON-Fe	62.4	67.9
PC-Fe	59.5	61.0
HEME-Fe	56.5	56.9
**SEM**	5.7	10.8
***P*-value**	0.625	0.620
	**Serum ferritin, ng/mL**	
CON-Fe	23.1	12.0
PC-Fe	27.9	10.2
HEME-Fe	37.2	15.6
**SEM**	6.7	3.3
***P*-value**	0.302	0.448
	**Serum total iron binding capacity, µg/dL**	
CON-Fe	168.0	384.7
PC-Fe	177.2	403.4
HEME-Fe	182.2	430.5
**SEM**	15.4	27.7
***P*-value**	0.783	0.221

^1^Number of observations per treatment group: Day 1—CON = 60, PC-Fe = 60, HEME-Fe = 56; Day 18—CON = 30, PC-Fe = 30, HEME-Fe = 28.

### Piglet Anemia Prevalence

**Figure 2.**
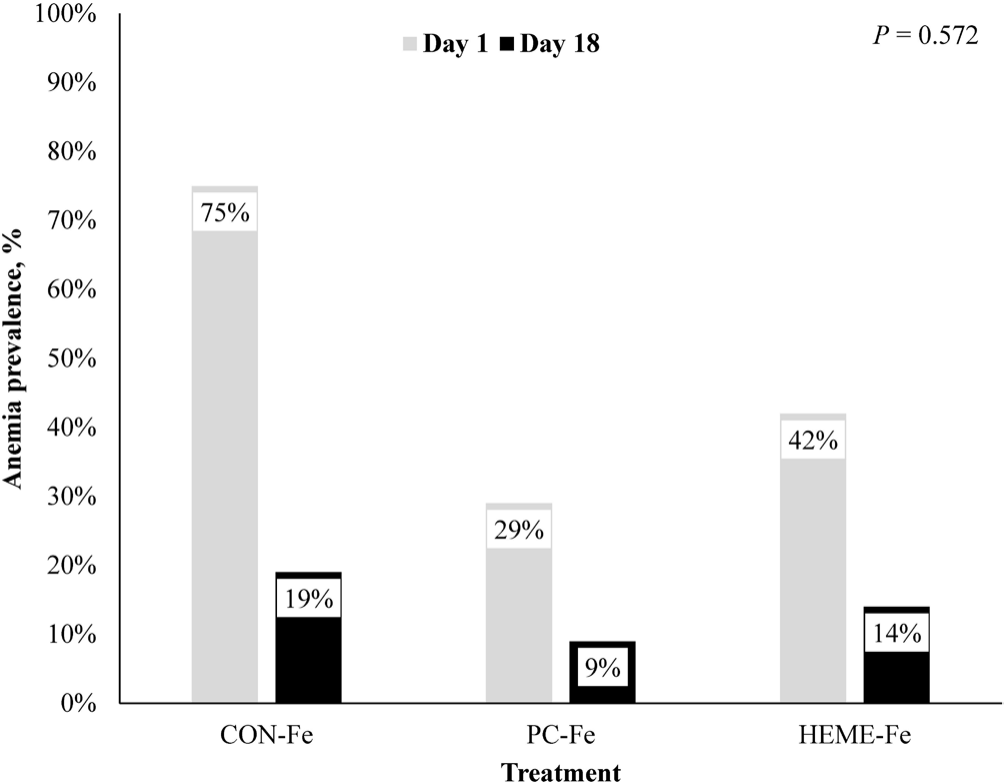
Piglet anemia prevalence on days 1 and 18 of age by iron source treatment Anemia is defined as hemoglobin < 10 g/dL.

The prevalence of anemia in offspring at birth (defined as Hb < 10 g/dL) was highest in the CON-Fe group (75%), followed by the HEME-Fe group (42%), with the lowest prevalence observed in the PC-Fe group (29%) (*P* < 0.001; Figure 2). By weaning, anemia prevalence was numerically lower in PC-Fe pigs (9%) compared to CON-Fe pigs (19%), with HEME-Fe pigs showing an intermediate prevalence (14%); however, these differences were not statistically significant (*P* = 0.122).

### Placental and Colostrum Mineral Content by Treatment

No differences were observed between treatment groups in the concentrations of trace minerals in placental tissue or colostrum (Table 7).

**Table 7.** Colostrum and placental tissue trace mineral content by iron source treatment

Item^1^	CON-Fe	PC-Fe	HEME-Fe	SEM	*P*-Value
Females, n	15	15	14	-	-
Colostrum, mg/L^2^					
Copper	5.7	5.5	5.1	0.4	0.520
Iron	1.4	1.4	1.2	0.1	0.201
Zinc	21.8	22.3	21.0	1.3	0.731
Placental tissue, mg/kg^3^					
Copper	8.7	8.6	8.3	0.8	0.901
Iron	269.5	337.6	285.9	33.2	0.331
Zinc	49.8	50.1	48.1	2.9	0.872

^1^Number of observations per treatment group: CON-Fe = 15, PC-Fe = 15, HEME-Fe = 14.

^2^Colostrum values (mg/L) are on an as-is basis.

^3^Placental tissue values (mg/kg) are expressed on a dry matter basis.

## DISCUSSION

This study evaluated the effects of three dietary iron sources—ferrous sulfate (CON-Fe), polysaccharide-complexed iron (PC-Fe), and heme iron (HEME-Fe)—on maternal and offspring iron status across a full reproductive cycle in highly prolific sows. While previous studies have reported beneficial effects of organic iron supplementation on piglet outcomes (Close, 1999 ; Peters and Mahan, 2008), few have comprehensively assessed how different iron sources influence maternal iron biomarkers throughout gestation and lactation. Given the widespread prevalence of gestational anemia in sows, identifying highly bioavailable iron sources that can improve maternal iron status and enhance fetal iron delivery is critical for optimizing both sow and piglet health outcomes.

While no differences were observed among treatments in sow BW, feed intake, farrowing duration, litter size, or piglet weaning weights, piglets born to PC-Fe-supplemented sows tended to be heavier at birth than those from CON-Fe sows, with HEME-Fe piglets showing intermediate birth weights. This trend is consistent with previous findings demonstrating improved birth weights with organic trace mineral supplementation (Close et al., 1999), likely due to enhanced maternal iron status supporting fetal erythropoiesis, placental function, and oxygen transport (Sangkhae et al., 2023). The intermediate response observed in the HEME-Fe group likely reflects the partial (50%) substitution of FeSO_4_ with HEME-Fe iron. Full replacement is necessary to fully understand the potential benefits of heme iron and permit more direct comparison.

Maternal hematological responses showed source-specific effects on iron metabolism indicators. HEME-Fe increased Hb at day 90 of gestation, a time of heightened erythropoietic demand as maternal RBC mass expands to support fetal development (Anderson et al., 1970; Matte and Girard, 1996 ). Heme iron is absorbed intact via specialized transporters such as heme carrier protein 1 and avoids the inhibitory interactions typical of non-heme iron (Le Blanc et al., 2012). Once internalized, heme is degraded by heme oxygenase, releasing ferrous iron for incorporation into Hb and other heme-containing proteins. This direct and highly efficient pathway likely explains the selective increase in circulating Hb observed in HEME-Fe sows. However, the effect was not sustained through late gestation and lactation, suggesting that 50% HEME-Fe inclusion may have been inadequate to meet the rising iron demands during this period.

In contrast, PC-Fe supplementation increased SF and tended to lower TIBC at day 30 of gestation. These shifts suggest enhanced iron storage capacity and improved iron transport efficiency (Smith et al., 1984). However, Hb concentrations remained similar to the CON-Fe levels in this group, indicating that iron was preferentially directed toward storage rather than immediate incorporation into erythrocytes. This contrasts with the response to HEME-Fe, which increased Hb without increasing SF, suggesting more efficient functional use of absorbed iron for erythropoiesis. The storage-focused response in PC-Fe sows may support the accumulation of maternal iron reserves early in gestation, potentially preparing the sow to meet the increased fetal iron demands that arise later in pregnancy.

Interpretation of hematological markers in gestating sows is complicated by physiological hemodilution. During pregnancy, plasma volume expands by approximately 20% (Matte et al., 1996), which can dilute Hb and PCV values and potentially obscure changes in iron status. Because iron is required to support the expansion of both red cell mass and plasma volume (Bothwell, 2000 ), Hb alone is an imperfect indicator of sufficiency. To account for this, a more comprehensive assessment of iron status should include a panel of biomarkers, such as SF, SI, and TIBC (Hytten, 1985). In the current study, while treatment effects were observed for Hb, SF, and TIBC, SI remained unchanged across treatments and timepoints. This likely reflects both its tight homeostatic regulation and its inherent biological variability. Serum iron levels are known to fluctuate in response to factors such as recent feed intake, time of day, and physiological stress (Dale et al., 2002; Hoppe et al., 2003; Wei et al., 2008), limiting its reliability as a standalone indicator of iron status.

The absence of treatment effects on RBC, WBC, PCV, or PLT levels suggests that the iron sources evaluated did not broadly influence hematopoietic or immune cell populations. In contrast, the selective increase in Hb concentrations in HEME-Fe sows highlights that Hb may be a more sensitive marker of iron utilization under physiological changes. These findings reinforce the importance of using multiple biomarkers to evaluate iron supplementation efficacy.

Although PC-Fe improved maternal SF at day 30 gestation, and HEME-Fe increased Hb at day 90 of gestation, neither effect was reflected in placental iron content. This is not unexpected given the tight regulation of uterine iron transport via proteins such as uteroferrin, which function independently of maternal iron status (Vallet et al., 2001). Furthermore, in highly prolific sows, placental iron transport capacity may be biologically capped, limiting additional iron deposition despite improved maternal stores (Kopeć et al., 2024).

Nonetheless, functional outcomes observed in this study—particularly reduced neonatal anemia—suggest that enhanced maternal iron status contributed to improved fetal iron status. Piglets from the PC-Fe group had the lowest proportion of anemic pigs at birth, followed by HEME-Fe, with the highest proportion of anemic pigs observed in CON-Fe litters. These differences support the link between maternal iron status during gestation and fetal outcomes. The improved iron storage observed in PC-Fe sows, as indicated by elevated SF in early gestation, likely enhanced the sow’s capacity to allocate iron to the fetus during the critical period of late gestation, when fetal iron accretion accelerates. Because iron must be mobilized from maternal stores and transferred across the placenta via tightly regulated pathways, higher maternal reserves may facilitate a more consistent and adequate fetal supply. Since Hb synthesis requires time even after postnatal iron injection (Heidbüchel et al., 2019), preventing anemia at birth is essential. Anemic piglets are at greater risk for impaired immune function, reduced growth, and increased pre-weaning mortality (Szudzik et al., 2018; Abbas et al., 2022), underscoring the importance of adequate maternal iron status—not only to meet the sow’s physiological demands, but also to ensure sufficient fetal iron accretion during late gestation.

The majority of piglet losses in this study occurred within the first week of life, emphasizing the critical role of adequate prenatal iron transfer in supporting early postnatal survival. The higher proportion of piglets weaned in the PC-Fe group and the intermediate proportion of piglets weaned in the HEME-Fe group compared to the CON-Fe group may have been driven, in part, by the lower proportion of piglets falling below the anemic threshold at birth. This aligns with previous studies showing that organic iron supplementation during gestation can improve neonatal iron status and reduce early-life mortality (Close, 1998, 1999) .

Although PC-Fe sows tended to wean a greater number of piglets than HEME-Fe sows, this difference likely reflects the numerically higher number of piglets born alive in the PC-Fe group. Because the proportion of pigs weaned was similar between these two groups, the difference in total pigs weaned appears to be a function of starting litter size rather than differences in pre-weaning survival. While it is possible that iron source may have influenced liveborn outcomes—particularly since dietary treatments began shortly after breeding—the observed numerical advantage of approximately 0.5 more total piglets born in the PC-Fe group compared to the HEME-Fe group may also reflect random variation in reproductive potential among sows. Importantly, sows were not stratified based on prior reproductive performance (e.g., total born, liveborn, or stillbirth rate), which may have contributed to baseline differences in litter size across treatments.

Iron source treatment had no effect on colostrum iron content. Although milk iron content was not assessed in the current study, postpartum mineral intake may influence the mineral composition of mature milk (Peters et al., 2010). However, previous research indicates that iron concentrations in colostrum and milk are generally unresponsive to maternal dietary iron source (Wang et al., 2014; Novais et al., 2016). Given the inherently low iron content of sow colostrum and milk (~1 to 2 mg/L) and the limited efficiency of neonatal iron absorption from these sources (Pond et al., 1965), prenatal iron accretion remains the primary determinant of neonatal iron status during the critical window between birth and postnatal iron injection.

A key limitation of the HEME-Fe treatment in the current study was the partial substitution of FeSO_4_ (50%), which was necessary due to practical constraints related to ingredient cost, formulation limits, and potential palatability issues. In contrast, PC-Fe fully replaced FeSO_4_. This discrepancy likely limited the ability of the HEME-Fe group to demonstrate its full biological potential. The intermediate responses observed in HEME-Fe sows and their offspring may reflect suboptimal heme iron inclusion rather than reduced intrinsic efficacy. However, this cannot be definitively concluded based on the current study design.

These findings should also be interpreted in the context of the elevated physiological demands of modern, high-prolificacy sows. Compared with earlier studies (Close et al., 1999; Peters and Mahan, 2008; Bertechini et al., 2012), sows in the current study carried larger litters, which substantially increases maternal iron requirements and exacerbates competition for iron between the sow and developing fetuses. As Mahan and Newton (1995) noted, higher levels of reproductive performance are associated with greater mineral losses, yet nutritional strategies have primarily focused on meeting energy, protein, and amino acid demands. Trace minerals, including iron, remain a critical yet often underemphasized component of sow nutrition, despite iron’s essential role in reproductive success (Fehse & Close 2000). Traditional inorganic iron sources such as FeSO_4_ may be insufficient to meet the elevated iron demands of modern sows. In contrast, more bioavailable sources, such as non-heme complexed iron or heme iron, may better support maternal iron status and fetal development under these high-demand conditions.

Finally, the absence of validated reference ranges for sow iron biomarkers limits the ability to accurately diagnose and manage subclinical iron deficiency. In humans, SF levels below 50 ng/mL are indicative of iron depletion (Guyatt et al., 1992), but no such thresholds exist for sows. Many sows in this study exhibited SF concentrations below this level despite not being classified as anemic by Hb. Without parity and reproductive stage specific reference intervals for SF, SI, and TIBC, accurate assessment of iron status remains challenging. Future research should focus on defining and refining these thresholds, and identifying optimal markers of iron status in sows for improvement of supplementation practices.

## CONCLUSIONS

PC-Fe supplementation effectively improved iron storage in sows and reduced the prevalence of anemia in piglets, likely contributing to the higher proportion of piglets weaned. In contrast, HEME-Fe increased maternal Hb concentrations but did not enhance iron storage to the same extent as PC-Fe. However, because the HEME-Fe treatment included only 50% of supplemental iron as heme, whereas PC-Fe fully replaced FeSO_4_, direct comparisons between the two sources should be interpreted with caution. Both organic iron sources (PC-Fe and HEME-Fe) were more effective than CON-Fe in reducing neonatal anemia, suggesting that organic forms of iron may offer superior protection against iron deficiency in offspring. Further research is warranted to establish physiologically relevant iron thresholds for sows at different reproductive stages and to refine supplementation strategies that optimize maternal status and support fetal development.
